# Histologic assessment of biliary obstruction with different percutaneous endoluminal techniques

**DOI:** 10.1186/1471-2342-4-3

**Published:** 2004-08-25

**Authors:** Michele Rossi, Vito Cantisani, Filippo Maria Salvatori, Alberto Rebonato, Laura Greco, Luigi Giglio, Giampiero Guido, Elisa Pagliara, Vincenzo David

**Affiliations:** 1 Department of Radiology, "S. Andrea" Hospital-II Faculty "La Sapienza" University, Rome,00100, Italy; 2 Department of Radiology, "UmbertoI" Hospital-I Faculty "La Sapienza" University, Rome,00100, Italy; 3 Department of Radiology, "Annunziata Civil Hospital"-Cosenza, 87100, Italy

**Keywords:** Biliary neoplasms, Bile ducts, biopsy, Bile ducts, cytology, Percutaneus-cholangioscopy

## Abstract

**Background:**

Despite the sophisticated cross sectional image techniques currently available, a number of biliary stenosis or obstructions remain of an uncertain nature. In these pathological conditions, an "intrinsic" parietal alteration is the cause of biliary obstruction and it is very difficult to differentiate benign from malignant lesions using cross-sectional imaging procedures alone. We evaluated the efficacy of different endoluminal techniques to achieve a definitive pathological diagnosis in these situations.

**Methods:**

Eighty patients underwent brushing, and or biopsy of the biliary tree through an existing transhepatic biliary drainage route. A subcoort of 12 patients needed balloon-dilatation of the bile duct and the material covering the balloon surface was also sent for pathological examination (balloon surface sampling). Pathological results were compared with surgical findings or with long-term clinical and instrumental follow-ups. Success rates, sensitivity, specificity, accuracy, confidential intervals, positive predictive value and negative predictive value of the three percutaneous techniques in differentiating benign from malignant disease were assessed.

The agreement coefficient of biopsy and brushing with final diagnosis was calculated using the Cohen's "K" value.

**Results:**

Fifty-six patients had malignant strictures confirmed by surgery, histology, and by clinical follow-ups. Success rates of brushing, balloon surface sampling, and biopsy were 90.7, 100, and 100%, respectively. The comparative efficacy of brushing, balloon-surface sampling, and biopsy resulted as follows: sensitivity of 47.8, 87.5, and 92.1%, respectively; specificity of 100% for all the techniques; accuracy of 69.2, 91.7 and 93.6%, Positive Predictive Value of 100% for all the procedures and Negative Predictive Value of 55, 80, and 75%, respectively.

**Conclusions:**

Percutaneous endoluminal biopsy is more accurate and sensitive than percutaneous bile duct brushing in the detection of malignant diseases (p < 0.01).

## Background

Bile duct dilatation and increasing jaundice are often onset symptoms of a number of either malignant or benign pathological conditions.

In most cases, common cross-sectional imaging techniques such as ultrasonography (US) or spiral-CT or cholangio-pancreatico-biliary magnetic resonance (MRCP) are highly capable of depicting the causes of obstruction by simply showing extrinsic masses in case of infiltrating tumors or benign causes of jaundice, like stones [1].

These non invasive techniques, however, can still yield uncertain results in some particular benign or malignant pathological conditions.

When stones are too small for example, or when bile duct dilatation is limited. For final confirmation or exclusion of endobiliary stones ERCP is unavoidable before the subsequent therapeutic steps.

There are also some pathological conditions where an "intrinsic" parietal alteration is the cause of biliary obstruction and it is very difficult to distinguish benign from malignant lesions using cross-sectional imaging procedures alone (figure 4 - 5).

A biliary dilatation may also occur after surgical interventions involving the biliary system, like bilioenteric anastomoses for gastric or pancreatic neoplasms or cholecystectomies. It is not always possible to distinguish between a recurrent disease or a fibrotic post-surgical stenosis, especially if it occurs early and if inflammatory alterations were already present during surgery.

A number of obstructions remains therefore unexplained because the aforementioned imaging modalities do not show any extrinsic compressing or infiltrating mass, nor a calcolous disease.

In these cases, only the intrahepatic bile ducts dilation and the level of the obstruction can be determined, but additional diagnostic procedures should be performed for a diagnosis of the real nature of the obstruction [2].

Patients with obstructive jaundice often undergo percutaneous biliary cholangiography and drainage (PBD) for decompression of the biliary tree.

Although relatively invasive, not even PBD allows to achieve a definitive differential diagnosis between malignant and benign pathologies.

Tissue sampling becomes therefore essential for the histological characterization of parietal alterations and for planning their appropriate treatment [3].

Bile cytology, although easily feasible either through a transhepatic or an endoscopic route, does not accomplish adequate pathological diagnoses in most cases [5-7].

In our study, three different endoluminal sampling techniques for the characterization of biliary strictures of uncertain nature have been reviewed and comparatively evaluated with the purpose of determining which one of them is the most accurate for differentiating between benign or malignant pathology.

## Methods

Our investigation was performed on a population of 80 patients who, from January 1992 to September 1999, underwent endoluminal brushing and/or biopsy for biliary stenosis or obstructions of uncertain nature. A retrospective evaluation of the efficacy of these techniques was carried out by comparing the pathological findings on one hand and radiological findings, clinical long-term follow ups, and/or post-surgical specimens, on the other.

Patient population included 43 men and 37 women, with a mean age of 62 years (range 37–87 years). All patients presented jaundice as a common symptom, weight loss was present in 20 cases (25%), and itching in 12 cases (15%).

At admission, all patients presented biliary dilation, but no masses were identifiable by imaging procedures such as US, CT, MRCP. ERCP had been performed in 23 patients with middle-low common bile duct stenosis but the nature of obstruction could not be determined nor a drainage could be placed.

Obstruction were located at the biliary bifurcation in 18 cases (22.5%), the common extrahepatic bile duct in 50 (62.5%), the right (2) and left (2) hepatic duct in 4 cases (5%)

Eight patients with bilioenteric anastomoses (10%) were also evaluated in order to determine the nature of the stricture. The patients came to the interventional unit needing for a biliary drainage. PBDs were performed using standard interventional techniques.[5]

The percutaneous approach was right lateral in all patients but 25 of them required also an anterior subxyphoid approach.

The procedures for cytological and/or histological samples were performed in any case 3–5 days after placement of the biliary drainage in order to operate on a decompressed biliary system.

One hundred and two endoluminal procedures were included in this study. Twenty-one patients underwent cytologic brushing only, 25 endoluminal biopsy only, and 22 both brushing and biopsy. A total number of 43 brushings and 47 biopsies were performed.

In 12 additional cases, we obtained cytological and tissue samples from an angioplastic balloon used for the bilioplasty of strictures of uncertain nature (balloon surface sampling) with techniques that will be described.

Sixteen patients underwent brushing only, because the cholangioscope was not available yet and five because the diagnosis of malignancy achieved with brushing was considered sufficient, thus not requiring further diagnostic workups to planning the appropriate therapy.

All the specimens were collected after PBD thus after translesional advancement of a 10 French catheter; in 12 particularly heavy stenosis a preliminary balloon dilatation was also accomplished.

**Figure 3:**
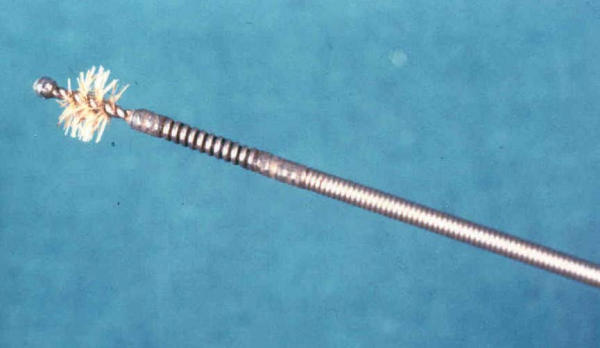
A metallic flexible probe with a cilindrical brush at the atraumatic tip, 5 mm in diameter 10 mm in length(Olympus Italia srl. Code number BC2029501) brush (approximately 1 cm in length and 5 mm in diameter)

Brushing was performed according to the following technical steps:

- the indwelling biliary drainage catheter was exchanged over a guide-wire with a 7/9-F. introducer sheath without valves at the proximal end ("peel-away", William Cook, Europe)

- a flexible probe (Fig. 3) with a cilindrical brush at the tip, 5 mm in diameter 10 mm in length (Olympus Italia srl. Code number BC20295010), was advanced through the sheath until it was beyond the lesion; the sheath was withdrawn to expose the brush, which was then pulled back and forth and rotated across the lesion several times under fluoroscopic control

- the brush was then carefully pulled back to be removed from the patient into the sheath, to avoid malignant spreading through the transhepatic tract

-samples were then placed on a glass slide, fixed with Sprayfix (Surgipath Medical Ind.; Illinois, USA), immediately submitted for cytological examination and stained by the standard Papanicolau technique.

**Figure 1:**
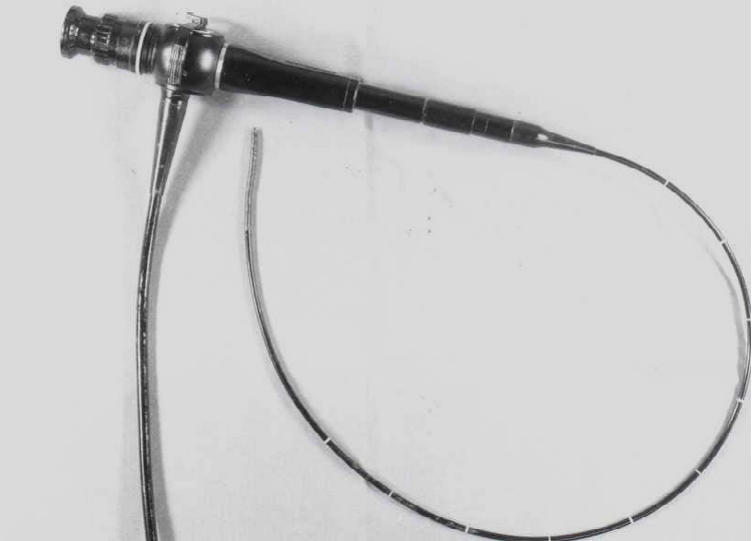
Flexible cholangioscope (5 mm in diameter) (Olympus, URF, type P, Japan)

**Figure 2:**
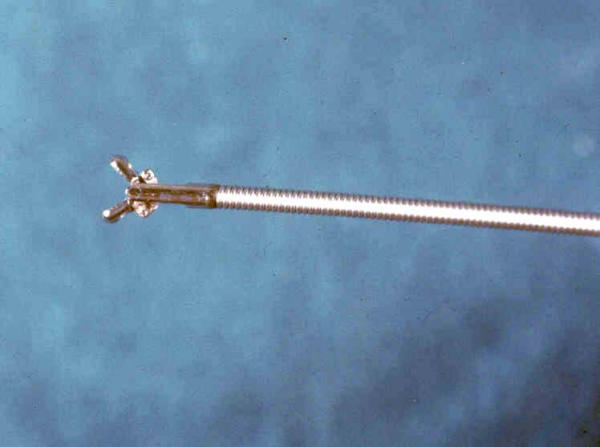
Alligator forceps (Olympus, FB 195X, Japan). The wire tip is open 25

Endoluminal forceps biopsy was performed under direct visualization with a 5-mm. cholangioscope (Olympus URF. Type P, Japan) (Fig. 1). Sometimes, the site of the lesion endoscopically visible did not correspond to the site of the stenosis fluoroscopically detectable; additional samples were therefore collected under fluoroscopic guidance, according to the following technique:

- the biliary catheter was replaced, over a guide, by a 18-F "peel-away" introducer sheath through which the cholangioscope was advanced until it was close to the lesion;

- a flexible forceps (5-F. Olympus, FB 185X Fig. 2) was passed through the working channel of the cholangioscope to obtain 3–4 specimens for each patient;

- the specimens were fixed in a 10%-solution of formaline and sent to the pathologist, included in paraffin, and dissected in slices of 2–4 microns at microtome; subsequently, they were stained by hematoxilyn-eosin, PAS and Mallory techniques;

- after completion of the biopsy procedures, all the patients underwent cholangiographic control checking for contrast material extravasation at the biopsy site and monitored for symptoms of hemobilia and/or bacteriemia.

All the patients were submitted to antiobiotic therapy before and during the three days following endobiliary procedures.

Twelve patients underwent preliminary bilioplasty because of the difficulties in advancing the drainage catheter across the biliary stricture. In this group, a different sampling was performed, based on the examination of those cells and/or tissue remained stuck to the angioplastic balloon after dilatation (balloon surface sampling):

- once deflated, the balloon (8–10 mm., Meditech, Boston, USA) was pulled back into the sheath and removed from the patient;

- the balloon was then placed in a saline solution with 10% of formalin, inflated and agitated several times to facilitate detachment of samples, which were then immediately delivered to the pathology laboratory.

Pathological specimens from 14 patients were compared with open surgery findings and post-surgical pathological reports. The pathologists, hystologists and cytologists, were blinded as to corresponding results.

The follow-up of the patients undergone to surgery stopped after the operation. Patients not candidate to surgery were treated with interventional procedures only such as bilioplasty, biliary drainage, interstitial radiotherapy and/or with chemotherapy or just a supportive therapy. This group of unoperated patients was followed-up on the basis of clinical and radiological data obtained by case-record reviews and by correspondence with their referring clinicians and general practitioners. The follow-up period ranged from 3 to 48 months. A minimum of 12 months of healthy negative cytological-histological diagnosis was necessary because either a clinically benign stenosing lesion or a cytohistological diagnosis negative for malignancy must be confirmed by a prolonged survival, as well as by clinical and radiological findings.

Calculations of success rate, sensitivity, specificity, positive predictive value, and negative predictive value for each technique were based on the number of biopsy procedures (n = 102) rather than on the number of patients (n = 80), (Table 1) since each biopsy was considered as a separate event The success rate is the percentage of biopsy procedures resulting in sufficient material for microscopic evaluation.

**Table 1:** Differentiation of biliary obstruction with different percutaneous endoluminal techniques

**Technique**	**N. PTS**	**SR**	**TP**	**TN**	**FP**	**FN**
Brushing	43 *	39/43	11	16	0	12
Biopsy	47 *	47/47	35	9	0	3
Balloon Brushing	12	12/12	7	4	0	1

SR= Success Rate; TP= True Positive; TN= True Negative; FP= false positive; FN= False Negative

*22 patients underwent either brushing and forceps biopsy.

Confidential Intervals (CIs) were determined for brushing and biopsy groups assuming a P value of .01 by using the Geigy Scientific Table (Geigy, Florence, 1984). The agreement coefficient between biopsy or brushing and final diagnosis was calculated using the Cohen's "K" value using SPSS 8.0 for Windows (SPSS, Chicago, Illinois, 1997).

## Results and discussion

A final diagnosis of malignant disease was confirmed in 56 (70%) cases and a final diagnosis of benign disease in 24 (30%) cases for an overall of 80 patients.

Final diagnoses in the malignant group included: cholangiocarcinoma (n = 31), adenocarcinoma (n = 6), metastatic adenocarcinoma (n = 5), pancreatic carcinoma (n = 13) and malignant endocrine tumor (n = 1). Final diagnoses in the benign group included: iatrogenic stenosis (n = 9), sclerosing cholangitis (n = 12) and primary (N = 2) and secondary biliary cirrhosis (N = 1).

Thirty-nine of the 43 brushing biopsies procedures were technically adequate for the diagnostic evaluation. Four cases were considered "poorly cellular" by the pathologists, with an overall success rate of 90.7% (Table 1). These 4 cases underwent endoluminal forceps biopsy within 15 days from the brushing.

Malignant cells were detected by brushing in 11 cases including: adenocarcinoma (n = 2), cholangiocarcinoma (n = 6), pancreatic carcinoma (n = 2), and metastatic adenocarcinoma (n= 1) (table 2).

**Table 2:** True positives

**Type of tumour**	**Brushing**	**Biopsy**	**Balloon-brushing**
Cholangiocarcinoma	**6**	**18**	**5**
Adenocarcinoma	**2**	**1**	**1**
Metastatic adenoca.	**1**	**5**	**-**
Pancreatic carcinoma	**2**	**10**	**1**
Neuroendocrine tumor		**1**	**-**

Of the 32 patients with negative findings for malignant cells, the 4 cases in whom the samples were considered "acellular" by the pathologists were excluded by the statistical analysis, as just mentioned. Hence, 16 out of 28 had a final diagnosis of benign disease confirmed by long-term clinical follow-up (true negatives) (Table 3) and 12 patients had a final diagnosis of malignancy (false negatives) (Table 4). Therefore, 11 true positives, 16 true negatives and 12 false negatives were obtained by cytological brushing, with an overall sensitivity, specificity, accuracy, PPV, and NPV in the detection of malignant diseases of 47.8, 100, 69.2, 100, and 57.1 % respectively (Table 1,5).

**Table 3:** True negatives

**Type of tumor**	**Brushing**	**Biopsy**	**Balloon-brushing**
Sclerosing cholangitis	**10**	**4**	**-**
Biliary cirrhosis	**3**	**2**	**-**
Iatrogenic strictures	**3**	**3**	**4**

**Table 4:** False negatives

**Type of tumor**	**Brushing**	**Biopsy**	**Balloon-brushing**
Cholangiocarcinoma	**7**	**-**	**1**
Adenocarcinoma	**2**	**1**	**-**
Pancreatic carcinoma	**3**	**2**	**-**

**Table 5:** Statistical analysis

**Technique**	**Sensitivity**	**Specificity**	**Accuracy**	**PPV**	**NPV**
Brushing	47.8% 28.10–69.66*	100% 87.30–100.00*	69.2%	100%	57.1%
Biopsy	92.1% 75.56–98.53*	100% 89.34–100.00*	93.6%	100%	75%
Balloon brushing	87.5% 52.30–99.96*	100% 64.31–100.00*	91.7%	100%	80%

* 99% C.I. (Geigy scientific tables)

Endoluminal forceps biopsy was performed in 47 cases. Thirty-five of these were interpreted as containing malignant cells (Table 2): cholangiocarcinoma (n = 18), pancreatic carcinoma (n = 10), metastatic adenocarcinoma (n = 5), adenocarcinoma (n = 1), and neuroendocrine tumor (n = 1). Nine of the 12 cases interpreted as containing inflammatory cells were confirmed by clinical and radiological follow-up as follows: sclerosing cholangitis (n = 4), iatrogenic stenosis (n = 3) and biliary cirrhosis (n = 2).

Three cases were erroneously interpreted as benign (false negative) but the clinical follow-up revealed 2 pancreatic carcinomas and 1 adenocarcinoma (Table 4). With 35 true positives, 9 true negatives and 3 false negatives, endoluminal forceps biopsy showed a sensitivity of 92.1%, a specificity of 100%, an accuracy of 93.6%, and a PPV and NPV of 100 and 75%.

CIs were reported in Table 5. "K" values for biopsy and brushing vs clinical/surgical final diagnosis were 0.613 and 0.404, respectively (Table 6).

**Table 6:** Cohen's Kappa value

	**Cohen's Kappa**	**Significance**
Brushing vs follow-up	0.404	0.001
Biospy vs follow-up	0.613	0.019

As previously discussed, 12 samples were obtained in as many patients with an angioplastic balloon after dilation of biliary strictures (balloon surface sampling). This technique demonstrated malignant cells in 7 (58.3%) cases and benign cells in 5 (41.7%). The final clinical diagnosis of the malignant group included adenocarcinoma (n = 1), cholangiocarcinoma (n = 5) and pancreatic carcinoma (n = 1) (Table 2). The final clinical diagnosis of the benign group included four iatrogenic stenosis (Table 3). In one case a sclerosing cholangitis was diagnosed by balloon brushing, but clinical follow-up and further investigations revealed a cholangiocarcinoma (Table 4). With this technique, we therefore had 1 false negative, 7 true positives and 4 true negatives, with a sensitivity, specificity, accuracy, positive predictive value and negative predictive value of 87.5, 100, 91.7, 100, 80%, respectively (Table 5).

Twenty-two patients underwent brush cytology together with endoluminal forceps biopsy. By excluding the 4 patients whose specimens were considered "acellular" and comparing each procedure with the clinical follow-up and surgical specimens, we obtained 12 concordant and 6 discordant diagnoses.

In the group of 12 concordant diagnoses, 6 benign diseases, such as primary sclerosing cholangitis (n = 3), iatrogenic stricture (n = 1) and biliary cirrhosis (n = 2) and 6 malignant diseases, such as cholangiocarcinoma (n = 3), pancreatic carcinoma (n = 2), and metastatic adenocarcinoma (n = 1) were included. All concordant diagnoses were confirmed by clinical or surgical follow-up. In the group of 6 discordant diagnoses, cholangiocarcinoma (n = 4) adenocarcinoma (n = 1) and pancreatic carcinoma (n = 1), were included. All the 6 malignancies misdiagnosed by brushing were correctly diagnosed by biopsy.

Transient hemobilia (spontaneously reversed from 1 to 12 hours after the procedure), was observed in 5/47 (10.6%) patients who had undergone biopsy.

In 4 of them, particular angulations of the access route was present and there were difficulties in negotiation of the stricture.

No major complications directly related to the brush cytology or the endoluminal forceps biopsy procedures occurred.

## Discussion

The evaluation of patients with biliary tract obstruction without evidence of any intrahepatic or extrahepatic growing mass has traditionally involved a variety of diagnostic imaging techniques [1]. Ultrasound and CT are often the initial diagnostic investigation to be performed and provide good information about the presence of biliary obstructions and the degree of ductal dilatation [8].

These imaging modalities, are however limited in depicting intraductal anatomy and, sometimes, in exactly determining the level and the cause of the obstruction [1,9]. Magnetic resonance cholangiography (MRC) is a non-invasive imaging modality providing a good visualization of the biliary. system. The sensitivity of MRC in the detection of choledocholithiasis has been reported as 90–100%, a comparable rate with that of endoscopic retrograde cholangiopancreatography (ERCP) [10-15].

The assessment of the level of obstruction also has been reported as highly accurate [16-19]. However, low-grade strictures or lesions causing biliary dilation may be missed by MRC [20]. The pathological characterization of presumed malignant strictures can be therefore, difficult, if not impossible, using noninvasive imaging studies alone in some intrinsic lesions causing biliary dilation. As a final diagnosis could radically affect further therapeutic choices histological, an histological characterization is required in the management of patients with biliary strictures [1].

Fine-needle percutaneous biopsy (FNPB) and fine-needle aspiration (FNA) has been reported poorly valuable in absence of a lesion clearly identifiable [21,22]. The tumor most frequently not identifiable as a true growing mass is cholangiocarcinoma and the differential diagnosis with primary sclerosing cholangitis is of somewhat importance [23]. The differential diagnosis between some carcinomas of the pancreatic head or small submucosal tumors of the ampulla and cholangiocarcinoma or inflammatory diseases, such as sclerosing cholangitis, can be very difficult in many cases. Patients with bilioenteric anastomoses after tumoral mass resection have a very complex local anatomic alteration that makes extremely difficult any radiological investigation searching for small recurrent neoplastic infiltrations [27].

Treatment protocols require pathological diagnoses for palliative or possibly curative therapy in almost all these types of conditions. In addition, the surgical technique itself can be different if a malignancy is present or not. Some Authors, when liver transplant is still indicated, suggest a large excision associated with gastrectomy, pancreatectomy and a transverse ascending colectomy [25].

Although the efficacy of this surgical approach is still under debate, the role of a preoperative diagnosis is extremely important.

Malignant cells surrounding the biliary ducts may continuously exfoliated into the bile, especially in the case of tumors which breack through the mucosa, and become available for the cytological examination when the bile is collected either percutaneously or by ERCP. Cytodiagnosis is easy to be performed, atraumatic in nature, with less potential risk and associated with relatively low charges. This technique has shown low sensitivity (15–28%) and accuracy (48–58%) rates, due to an early cellular degeneration after bile collection and to "poorly cellular" specimens. In addition, some pathological aspects can affect the efficacy of the procedure, such as in case of lesions extrinsically compressing the bile duct wall without a complete transmural infiltration or an adequately wide mucosal disruption [22]. Endoluminal brush cytology was successfully performed either percutaneously, by Radiologists, or endoscopically, by Gastroenterologists [26-28]. In our experience, brush cytology showed a high specificity but low sensitivity and accuracy rate in the detection of malignant diseases. Technical limits were mostly represented by "poorly cellular samples". In our study, in fact, 4 cases (9.3%) were considered "poorly cellular" by the pathologists. In addition to these technical problems, similarly to bile cytology, we have to consider some morphological aspects that can negatively influence the sensitivity of this technique.

According also with our cholangioscopic experience, biliary tumors may remain intramural causing an annular constriction of the biliary duct [21,29] without complete transmucosal infiltration, and this condition can mimic sclerosing cholangitis and render brush cytology ineffective. In light of the fact that there is a frequent relationship between sclerosing cholangitis and malignant strictures, the cytological differentiation between inflammatory and malignant changes can be extremely difficult. In addition, these tumors are frequently so well differentiated that their identification as "malignant" can be, even histologically, difficult [29,30]. These are the theoretical explanations that we can give to the fact that cytological diagnosis of cholangiocarcinoma yielded a relatively low sensitivity, verified in our study as well as in the literature [1,21-23,26,30]. At endoscopic sampling, cholangiocarcinoma has a higher sensitivity [28]

In this review only patients not suitable for ERCP or coming from a failed drainage or other type of retrograde endoscopic intervention were evaluated, thus the percutaneous and endoscopic techniques can not be compared. Whenever possible especially if skilled endoscopists are available, retrograde approach could still to be considered the first step, for its potentially high sensitivity especially if repeated sampling are performed [27,28]

Percutaneous brush cytology, if compared with bile transendoscopic cytodiagnosis, has the potential risk of a malignant spreading of cells through the transhepatic tract. To overcome this risk, in our opinion, the brush should be pulled back and removed from the patient into the introducer sheath. With this technique, in fact, no spreading along the transhepatic tract was observed in our malignant patients at imaging follow-up. Brush cytology is a easy, safe and at relatively low-cost procedure, similarly to bile collection. A single sampling however has a low possibility of detecting a malignancy. The results can improve with multiple samples. Three consecutive negative samples decrease the probability of a malignancy from more than 55% to less than 5% [30]. The absence of false positives in our and others, series [2] means that an intraductal biopsy has no purpose when an exfoliative cytology is positive. Meanwhile, in presence of a negative cytology, other techniques such as percutaneous FNA and endoluminal forceps biopsy should be mandatory. Fine needle aspiration performed either percutaneously or endoscopically has some technical advantages over endoluminal brushing in those lesions extrinsically compressing the biliary duct without deeply infiltrating the ductal wall, since the inner epithelial layer is not involved [21]. Most of these patients with biliary obstruction, especially with high lesions, however, in our Hospital, usually undergoes percutaneous biliary drainage in the early phases of their clinical work-up. A percutaneous FNA would represent an adjunctive interventional procedure that could be avoided by using the transhepatic route, already available. We had adequate percutaneous biopsy specimens in epithelial lesions, such as cholangiocarcinoma, and inadequate specimens in those lesions with inflammatory and/or necrotic changes. The dense fibrotic and scirrus reaction associated with pancreatic carcinoma may result in poor biopsies specimens [31]. In addition, pancreatic carcinoma is often associated with pancreatitis, necrotic cellular debris and a dense fibrotic reaction which can further contribute to the disappointing results obtained with even aggressive percutaneous biopsy techniques [32].

The differentiation degree of a tumor can affect the accuracy of the histological classification. In those cases of extrahepatic and periampullary biliary tumors, in fact, usually highly undifferentiated, the histological characterization can be difficult, although a generic diagnosis of malignancy can be made.

Potential complications due to forceps biopsy, such as disruption of the ductal wall with consequent bile leak and bleeding, cholangitis and pancreatitis, are reported [33].

Endoluminal forceps biopsy especially in patients with hemobilia or cholangitis, should therefore be performed in the remission phase of the disease, and, in any case, at least 5 days after biliary drainage, to avoid the complications related to manipulations into the biliary tree. No major complications were observed in this series, neither from percutaneous brushing nor from forceps biopsy. A transient hemobilia was observed in 5 patients who had undergone biopsy (10.6%). This series, including part of a previously analyzed smaller population [33] confirms that percutaneous endoluminal forceps biopsy has a very high sensitivity (92.1%), specificity (100%), accuracy (93.6%), PPV (100%), and NPV (75%) in the detection of malignant diseases (Table 2). The higher accuracy of biopsy over brushing is very clear, especially analyzing the data obtained in those patients in whom both techniques were performed. On the other hand, biopsy presents some disadvantages, such as the higher costs of the equipment and the difficult trackability across tight strictures or acute angles of the biliary ducts.

A high diagnostic value was proven by the simple examination of tissue samplings coming from balloon dilatation of biliary or bilio-enteric anastomotic strictures (balloon surface sampling). Although the number of cases reported in this series is relatively small, it should be considered that it was possible to distinguish benign from malignant diseases in almost all the cases. Our suggestion, therefore is to associate a tissue collection from the balloon to bilioplasty, as a standard procedure when screening for malignant pancreatobiliary diseases is required. In this way. it is possible to save time and avoid risks related to further endobiliary procedures.

## Conclusion

Patients with obstructive jaundice, who are candidates for biliary drainage, often come to the interventional radiologist still without a definitive diagnosis of the real nature of their disease, even after high-level cross-sectional imaging procedures, such as abdominal MR, MRCP or abdominal spiral-CT. When the clinical diagnosis needs to be histologically confirmed for further therapeutic choices, the transhepatic route can be successfully used for the intraductal sampling. Forceps biopsy is highly accurate under cholangioscopic guidance. As an alternative, repeated brushings can be performed under fluoroscopy. If a balloon blioplasty, for any reason, is performed, it is advisable to collect the tissue fragments on the balloon surface and send them for pathological evaluation.

## Competing interests

None declared.

## Authors' contributions

MR carried out the interventional procedures, participated in the design of the study and drafted the manuscript, FMS carried out the interventional procedures, VC defined the design of the study and performed the statistical analysis, LG collected the results of the procedures, LG participated in the histological analysis, AR participated in the design of the study and drafted the manuscript,, GG participated in the imaging evaluations, EP performed the patients follow-up, VD supervised the drafting of the study

**Figure 4:**
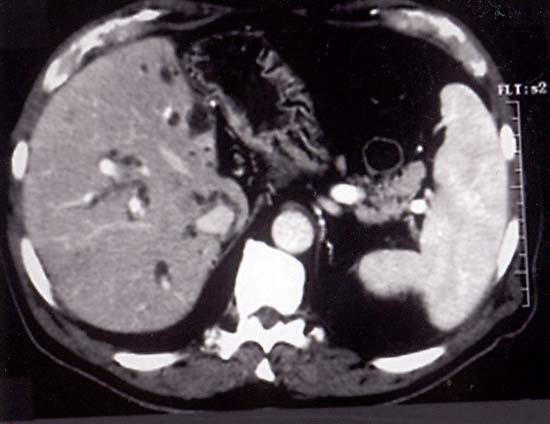
A 58 year old man, who 8 years ago underwent left hepatectomy and cholecistectomy, for complicated intrahepatic biliary stones, presented with jaundice and weight loss. Enhanced CT scan showed marked intrahepatic biliary dilation.

**Figure 5:**
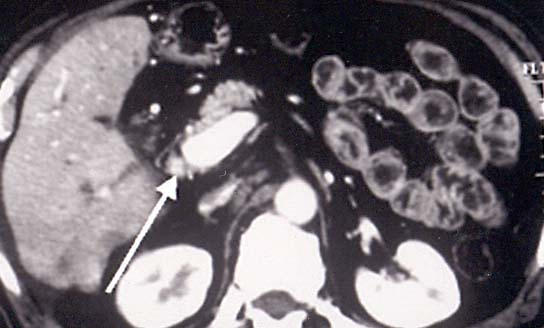
Same patient as fig 4, at lower level, although, an extrinsic mass was not detected, the lumen of CBD appeared replacement by soft tissue mass density (arrows).

## Pre-publication history

The pre-publication history for this paper can be accessed here:


